# Radiofrequency ablation of thyroid nodules: prospective cost-effectiveness analysis in comparison to conventional thyroidectomy

**DOI:** 10.20945/2359-3997000000411

**Published:** 2021-11-11

**Authors:** Marcelo Soares Schalch, Anna Carolina Novais Costa, Rafael Pereira de Souza, Filipe Lamounier Barros Guerra, Roberta Guerreiro, Rafael De Cicco

**Affiliations:** 1 Instituto do Câncer Arnaldo Vieira de Carvalho Departamento de Cirurgia de Cabeça e Pescoço São Paulo SP Brasil Departamento de Cirurgia de Cabeça e Pescoço, Instituto do Câncer Arnaldo Vieira de Carvalho (ICAVC), São Paulo, SP, Brasil

**Keywords:** Radiofrequency ablation, partial thyroidectomy, thyroid nodule

## Abstract

**Objective:**

The objective of this study is to compare the total costs of surgery and radiofrequency (RF) ablation for the treatment of benign thyroid nodules.

**Materials and methods:**

This is a prospective randomized study comparing cases treated with US-guided RF ablation (cases) and surgery (control). They were selected and allocated to groups (thyroidectomy or radioablation) by permuted block randomization in blocks of five cases each.

**Results:**

Five cases of RF Ablation were compared with five cases of thyroidectomies conducted in the same period. Similar complication rates were observed in both groups. Shorter operating time and hospital stay were observed for the RF group. In the evaluation of the total cost between procedures, radioblation represented 76% of the cost of partial thyroidectomy.

**Conclusion:**

This study demonstrated that radioablation has a competitive cost, making it an effective alternative in the treatment of benign thyroid nodules.

## INTRODUCTION

Thyroid nodules are a common finding in clinical practice (1). The prevalence of thyroid nodules identified by palpation alone on physical examination varies from 4% to 7%, whereas autopsy studies identified a prevalence of clinically nonpalpable nodules of up to 65% (2,3). With the advancement of imaging tests, including ultrasound, tomography, nuclear magnetic resonance and positron emission scanning (PET-CT), it has become more common to identify previously undiagnosed nodules (4,5). Ultrasound (US) can detect clinically nonpalpable nodules in 20% to 76% of the adult population (6,7).

With the emergence and increased availability of and access to US, we have observed an increase in the identification and diagnosis of thyroid nodules. As a result, the challenge of finding the best treatment with the lowest number of complications and sequelae for the patient that is also economically viable has arisen.

The current indications for the treatment of benign thyroid nodules, according to the guidelines of the American Thyroid Association (ATA) (8), include bulky nodules larger than 4 cm in diameter that cause both compressive and aesthetic symptoms. The patient’s desires regarding the treatment modality should also be taken into account (9).

The treatments that were commonly used over the last decades were surgery, hormone suppression or clinical observation. Surgical treatment is associated with some complications, such as recurrent laryngeal nerve injury and its consequences (0.3% to 1.7%), hypoparathyroidism with permanent hypocalcemia (1.3% to 3%), permanent scarring of the neck and hypothyroidism, which occurs in 5% to 49% of lobectomy cases and 100% of total thyroidectomy cases (3,10,11). Although such complications are rare in procedures performed by high-volume surgeons, they cannot be neglected. With this therapeutic modality, there is also the fear of possible cervical hematoma, severe complications and the need for additional surgery. There is also a need for hospitalization and general anesthesia (7,12). Another option is clinical treatment with hormonal suppression; however, the efficacy of this treatment is controversial, and there is no evidence of obvious effects on nodule growth inhibition or size reduction (7,12).

Ablation with ethanol for the treatment of benign thyroid nodules was also effective but is associated with high recurrence rates (5% to 25%) (13-15); it is indicated mainly for predominantly cystic nodules, since the solid components are considered more resistant to diffusion and the vascularization of solid nodules favors ethanol drainage, thus limiting the success of the procedure in predominantly solid nodules (16).

Clinical observation in patients with large, symptomatic and predominantly solid thyroid nodules carries the probability that the nodule will progress and the symptoms will worsen, in addition to not providing relief for the already present symptoms.

The desire to avoid the anterior neck scar has led to the development of remote-access thyroidectomy techniques, which have surgical complication rates that are the same as those of conventional surgery. However, this surgical method depends on the location and size of the thyroid nodules, and not all nodules are suitable for this type of treatment (17,18).

More recently, radiofrequency (RF) ablation has emerged as a minimally invasive method that preserves thyroid function and offers satisfactory results in terms of thyroid nodule volume reduction and without recurrence or the need for additional surgery (19).

Like most medical innovations, its cost should be taken into account, and high costs may be a barrier to the implementation of RF, especially in developing countries such as Brazil. However, there are no data in the literature that support this concern, nor are there studies that compare the costs and effectiveness of surgery versus RF ablation.

## MATERIALS AND METHODS

The objective of this study is to compare the total costs of surgery and RF ablation for the treatment of benign thyroid nodules.

This is a prospective randomized study comparing cases treated with US-guided RF ablation (cases) and surgery (control). Patients from the Department of Head and Neck Surgery of Arnaldo Vieira de Carvalho Cancer Institute (ICAVC, for its acronym in Portuguese) were selected and allocated to groups (thyroidectomy or radioablation) by permuted block randomization in blocks of five cases each.

This study was approved by the research ethics committee of Arnaldo Vieira de Carvalho Cancer Institute (CAAE nº 19516619.2.0000.5471). Only patients with thyroid nodules with benign cytology prior to treatment were included in the study. The inclusion criteria were patients with thyroid nodules diagnosed as benign with fine-needle aspiration biopsy (FNAB) according to the Bethesda classification (8); predominantly solid (>50%) thyroid nodules on US; nodules larger than 3 cm; nodules causing aesthetic problems or compressive symptoms; and the desire to receive treatment.

The exclusion criteria were the presence of nodules that were indeterminate or suspicious for malignancy; nodules that were smaller than 3 cm and asymptomatic; clinical comorbidities that contraindicated surgery; and patient refusal to participate in the study. After randomization, the patients were informed about the proposed treatment for their group and completed the informed consent form. No patient refused the proposed treatment.

The variables collected related to the patient (sex, age, comorbidities), disease (nodule size at 0, 30, 60 and 90 days) and cost (operating room time and procedure duration, length of hospital stay, total hospital cost, type of anesthesia and cost of materials used).

To perform the cost analysis, the actual cost tables of our service (ICAVC) were used. These include the costs of daily stays, medications, surgery, pathology and materials adjusted according to the month of the procedure. For standardization, the SIMPRO table (materials and medications) number 128, in circulation in June/July 2020, was used. The cost of procedures and doctor’s fees were calculated according to the cost table of the Hierarchical Brazilian Classification of Medical Procedures (CBHPM, for its acronym in Portuguese; 5^th^ edition). In addition, the cost of special materials for RF ablation treatment were established by the manufacturer and supplier of Surgical Line^®^ equipment.

Descriptive statistical analyses were performed. Additionally, comparisons between the groups were made using the chi-square test for categorical variables and Student’s t-test for continuous variables.

## RESULTS

The mean age of the patients in both groups was 46.4 years, with no significant difference between groups (p = 0.79). All patients in this study were female, which increased the homogeneity of the groups (Table 1).

**Table 1:** Distribution of the variables by group

Variables	Thyroidectomy	Radiofrequency Ablation	p
Age	47.6	45.2	0.79
Gender			
Operation time – mean	90	71.4	0.29
Operating room time – mean	172	90	0.01
Complications			
No	3	3	0.51
Edema	2	1	
Vocal cod palsy	0	1	
Nodule diameter – mean	39.4	40.6	0.72
Fine needle aspiration biopsy	II (100%)	II (100%)	
Hospital stay – hours	26.2	8.6	0.01
Cost of material – charged by hospital	416.69	225.42	0.01
Cost of material – SIMPRO	2137.93	1419.11	0.02
Cost of hospital stay – charged by hospital	667	171.99	0.01
Cost of hospital stay – SIMPRO	413.74	146.27	0.01
Medical costs – CBHPM	1273.66	1070	0.01
Total cost CBHPM + SIMPRO	3825.33	2661.1	0.007

The most frequently observed comorbidity was systemic arterial hypertension, which affected four of the five patients in the case group and one patient in the control group. This was followed by asthma, which affected one patient in the case group and two in the control group.

Regarding preoperative symptoms, compressive symptoms were present in one patient in each group, and all other patients were asymptomatic.

**Figure 1:**
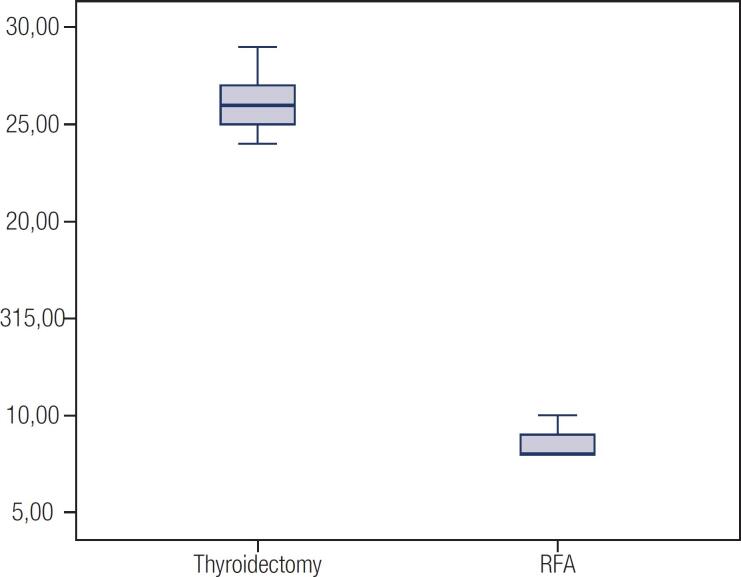
Length of hospital stay (hours) for each group.

The two groups differed widely in operating room time. The RF ablation group had a mean procedure duration of 71.4 minutes, ranging from 40 to 118. In the partial thyroidectomy group, the surgery time ranged from 60 to 120 minutes, with a mean of 90 minutes. RF ablation resulted in a 20.6% reduction in operating room time (p = 0.01). The mean length of hospital stay in the RF ablation group was significantly lower (17.6 hours) than that of the thyroidectomy group (26.2 hours) (p = 0.01) (Table 1, Figure 1). There was no need for hospital stay in the case group. It is important to emphasize, however, that admission in our service is routinely performed the night before procedure. This practice ensures floor vacancy for the patient to undergo the procedure. In the RF group, three patients (60%) were hospitalized the night before, while in the partial thyroidectomy group, four (80%) were hospitalized the day before. This practice resulted in an overall increase in length of hospital stay in both groups.

The mean nodule size in the RF ablation group was 37.57 mm, whereas in the thyroidectomy group, it was 39.4 mm; the FNAB findings for all nodules were as benign according to the Bethesda classification (Table 1).

In the RF ablation group, 60% of patients had no postoperative complaints, and the same proportion experienced no complications. One patient (20%) complained of neck “swelling” and the postoperative complication of local edema, one patient (20%) complained of paresthesia in the right arm, and one patient (20%) developed a nonexpanding and nonpulsatile hematoma. All complications were self-limited and conservatively treated, and none resulted in prolonged hospitalization.

In the thyroidectomy group (surgical), 60% had no complaints during the postoperative period. One patient presented with hoarseness and unilateral vocal fold paralysis, and one patient complained of “weak voice at the end of day”. All complications were self-limited, did not require prolonged hospitalization and were treated conservatively.

None of the patients had symptoms of hypoparathyroidism, and none of those who underwent PTH testing during the postoperative period had a PTH level lower than 27 pg.

**Figure 2:**
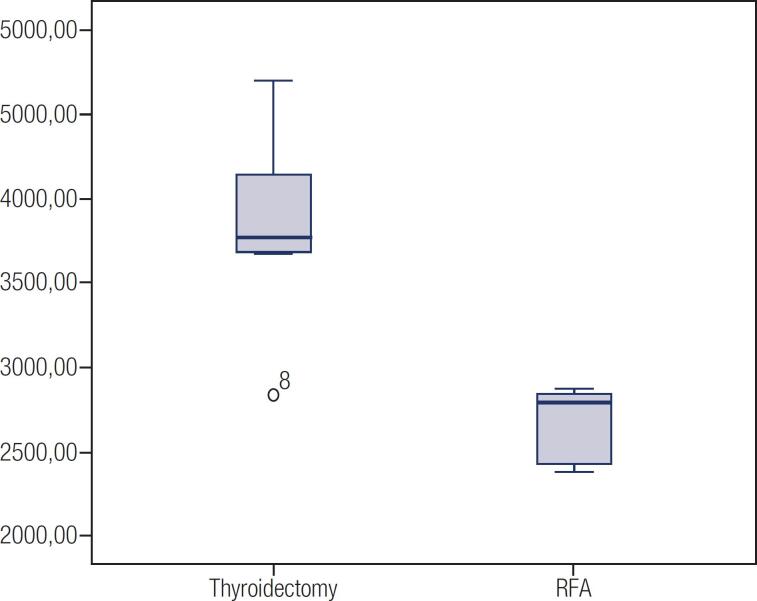
Standardized costs of materials and medications (CBHPM/SIMPRO).

To ensure adequate standardization, we used the CBHPM and SIMPRO tables. The patients in the RF ablation group did not require daily hospitalization and had a total hospital cost of R$ 146.27 per patient based on the CBHPM table. In contrast, the average hospitalization cost of the thyroidectomy group (also based on the CBHPM table) was 2.82 times higher (p = 0.01), totaling R$ 413.74 per patient (Table 1, Figure 2).

A difference was also found in the cost of materials and medications. According to the SIMPRO table, the average cost of RF ablation was R$ 1,419.11, while for thyroidectomy, it was R$ 2,137.93, which was 1.50 times higher (p = 0.02). The cost difference regarding medications is mainly justified by the fact that RF ablation group underwent procedure under sedation and local anesthesia, while the partial thyroidectomy group underwent general anesthesia. When the costs of orthoses, prostheses and special materials (OPSM) [intraoperative nerve monitoring – R$ 14,500.00; harmonic shears – R$ 5,634.36; radiofrequency ablation needles – R$ 7,000.00; the cost of radiofrequency ablation needles was determined by the supplier (Surgical Line^®^), and that of the other materials was based on the SIMPRO table] were included, the average total cost of materials for patients in the RF ablation group was R$ 8,419.11, while for the thyroidectomy group, it was R$ 10,137.93. Although we do not use OPSM in our service, we decided to add the cost, once we believe this material is widely used in practice, in our country, among private and even public hospitals.

Pathology/freezing cost of R$ 0 for patients undergoing RF ablation since there were no anatomical-pathological specimens, while in the control group, this cost averaged R$ 344.16 per patient (Table 1).

Finally, according to the CBHPM table, doctors’ fees also cost less for the RF ablation group (R$ 1,070) than for the thyroidectomy group (R$ 1,273.66), which had a cost that was 1.19 times higher (p = 0.01).

When all costs were summed, it was determined that the cost of RF ablation was 40% of the cost of partial thyroidectomy. The average cost of R$ 9,661.10 per RF ablation patient was lower than the average cost of R$ 23,959.69 per patient undergoing thyroidectomy. In addition, the actual costs paid by the institution also differed between the procedures.

## DISCUSSION

RF ablation of thyroid nodules is a relatively new procedure (19). It is a minimally invasive treatment that has been shown to be effective and to have a low risk of complications. This therapeutic modality has the advantage of being minimally invasive, effective, safe, economical and aesthetically satisfactory, which enables early recovery of a normal lifestyle and work activity in addition to having a lower rate of complications. This modality preserves thyroid function in most cases, and the rate of RF ablation patients who later require surgery to control symptoms was zero in a recent study (7).

The efficacy of RF ablation was demonstrated not only by the absence of clinical problems but also by the reduction in volume that was observed on US at the 6-month follow-up after the procedure. RF ablation is effective for nonfunctioning, autonomous or malignant thyroid nodules, regardless of their solid component (20-22).

According to Jeong and cols. (21), the reduction in the mean volume of 140 nonfunctioning thyroid nodules observed at the 6-month follow-up was 84.79% after one or more than two RF ablation sessions (mean = 1.4). When the nodules were grouped into mainly cystic, mixed and mainly solid nodules, the volume reduction was significantly greater for the solid nodules than for the other types at the 1-month follow-up. However, at the 6-month follow-up, there was no significant difference in volume reduction among the three types. Tang and cols. (3) observed a complete ablation rate of thyroid nodules of 98.95% with RF.

Regarding thyroid function, cases of hypothyroidism after RF ablation have been reported. The cause of hypothyroidism is not yet clear (23-26). Some studies have reported transient or permanent hypothyroidism in some patients after RF ablation. Although thyroid function seems to only rarely be influenced by RF ablation, it is not clear whether RF ablation affects thyroid function in patients with bilateral benign thyroid nodules.

Thus, the use of US-guided percutaneous RF ablation to treat benign thyroid nodules has the advantages of definitive efficacy, significant reduction of lesion volume, high nodule regression rate, reduced damage to the surrounding normal tissue and a low complication rate (most complications are reversible and temporary). There is no surgical incision, and the method can maximally satisfy patients’ aesthetic demands; therefore, RF ablation offers an adequate first-choice treatment for benign thyroid nodules and, in some cases, it can replace surgery as a treatment for benign thyroid nodules.

The debate on the viability of RF ablation as a therapeutic option for thyroid nodules reflects concerns about the increased cost of this modality compared to the current gold standard treatment, especially in developing countries such as Brazil. However, our study demonstrated good cost-effectiveness in the case group, with the RF ablation representing 81% of the cost of the current gold-standard treatment. We cannot disregard the cost of the device, which is R$ 7,000.00, according to the distributor. This is higher than the total hospital cost of both studied procedures, according to the referenced tables. However, we also cannot disregard the fact that most thyroidectomies, especially those performed in the context of private health insurance, currently use high-cost materials such as nerve monitoring systems, hemostats and harmonic shears, the costs of which were included in the results of the study.

The standard treatment for benign thyroid nodules is surgical, in the form of partial or total thyroidectomy. This treatment has great therapeutic efficacy, offers the possibility of evaluating the specimen anatomically and pathologically and is well accepted by the medical community. However, it is not without complications.

The RF ablation technique, which was recently introduced for the treatment of thyroid nodules, offers the advantages of not requiring general anesthesia, early hospital discharge and absence of a neck scar, with well-established results. Furthermore, the use of US-guided percutaneous RF ablation to treat benign thyroid nodules not only has the advantages of definitive efficacy, significant reduction of lesion volume, high nodule regression rate, reduced damage to surrounding normal tissue and a low rate of complications but also lower costs than surgical treatment. Thus, RF may be adequate as the treatment of choice for benign thyroid nodules and, in some cases, may replace the surgical treatment of benign thyroid nodules.

In conclusions, the study showed that despite being a newly incorporated technology, RF ablation has a competitive cost, making it an effective option for the treatment of benign thyroid nodules.
